# The optimal number of personnel for good quality of chest compressions: A prospective randomized parallel manikin trial

**DOI:** 10.1371/journal.pone.0189412

**Published:** 2017-12-21

**Authors:** Syunsuke Yamanaka, Ji Young Huh, Kei Nishiyama, Hiroyuki Hayashi

**Affiliations:** 1 Department of Emergency Medicine, University of Fukui Hospital, Fukui, Japan; 2 Department of Family Medicine, Adventist Medical Center, Okinawa, Japan; 3 Department of Trauma and Critical Care Center, National Hospital Organization Kyoto Medical Center, Kyoto, Japan; 4 Department of Family Medicine, Family Medicine, University of Fukui Hospital, Fukui, Japan; Waseda University, JAPAN

## Abstract

**Background:**

Long durational chest compression (CC) deteriorates cardiopulmonary resuscitation (CPR) quality. The appropriate number of CC personnel for minimizing rescuer’s fatigue is mostly unknown.

**Objective:**

We determined the optimal number of personnel needed for 30-min CPR in a rescue-team.

**Methods:**

We conducted a randomized, manikin trial on healthcare providers. We divided them into Groups A to D according to the assigned different rest period to each group between the 2 min CCs. Groups A, B, C, and D performed CCs at 2, 4, 6, and 8 min rest period. All participants performed CCs for 30 min with a different rest period; participants allocated to Groups A, B, C, and D performed, eight, five, four, and three cycles, respectively. We compared a quality change of CCs among these groups to investigate how the assigned rest period affects the maintenance of CC quality during the 30-min CPR.

**Results:**

This study involved 143 participants (male 58 [41%]; mean age, 24 years,) for the evaluation. As participants had less rest periods, the quality of their CCs such as sufficient depth ratio declined over 30-min CPR. A significant decrease in the sufficient CC depth ratio was observed in the second to the last cycle as compared to the first cycle. (median changes; A: −4%, B: −3%, C: 0%, and D: 0% p < 0.01).

**Conclusions:**

A 6 min rest period after 2 min CC is vital in order to sustain the quality of CC during a 30-min CPR cycle. At least four personnel may be needed to reduce rescuer's fatigue for a 30-min CPR cycle when the team consists of men and women.

## Introduction

Chest compressions (CCs) play a vital role in successful cardiopulmonary resuscitation (CPR)[1]. According to American Heart Association (AHA) 2015 guideline, high quality of CCs demand several conditions; Hand position should be two hands on the lower half of the sternum, chest compression should be performed at a rate of 100-120/min, full recoil of chest should be performed after each compression and minimizing interruptions[1]. CCs with an adequate depth has been recognized as an important aspect of high-quality CPR for survival and for favorable neurological outcomes[2]. Compression depth has been changed to at least 5 cm but should not exceed 6 cm in 2015 AHA recommendations from at least 5 cm in 2010[1]. The definition of appropriate rate of CCs was also changed to 100-120/min in 2015 from at least 100/min in 2010. In recent observational study, increased median duration of resuscitation, longest 25-mins, had a higher likelihood of return of spontaneous circulation during in-hospital cardiac arrest.[3] This study suggested the longer duration of CPR could improve survival rate in–hospital cardiac arrest, however, to maintain correct compressions with sufficient depth is considerably difficult throughout the course of long CPR. [4, 5] Many previous studies have proven that rescuer fatigue negatively affects CC depth especially during the longer CPR.[4, 6, 7] Although AHA recommends to change chest compression person in every 2 min cycle to maintain high quality CPR, to our knowledge, no previous study has illustrated the sufficient number of personnel in total required to perform CCs taking into account minimal rescuer fatigue in 30 min CPR. The aim of our study was to ascertain the sufficient number of personnel needed for CCs with adequate depth during 30 min CPR.

## Materials and methods

### Trial design and ethical considerations

This study was designed as a prospective, randomized, parallel manikin trial conducted from April 1, 2015, through December 1, 2016. The ethics board of the Faculty of Medical Sciences, Fukui University approved this study. Informed consent was obtained from all participants for this study before the trial in the form of documents. The clinical trial number was UMIN (UMIN: University Hospital Medical Information Network) 000027112.

### Participants

We recruited the following participants: medical students from the University of Fukui, junior residents (Post graduate year one and two), nurses and staff doctors of Emergency Department from Fukui University Hospital and emergency medical services (EMS) personnel from Fukui City Fire Department. These subjects are expected to participate as rescuers to perform CCs during CPR.

### Study setting

We provided participants a 30-min lecture about appropriate chest compression and a 20-min chest compression practical training with manikin according to the 2010 AHA guideline[8]. All participants took a brief knowledge and skill examination after the training. They needed to repeat the test until they got full score in order to begin the trial session of chest compression. Participants were asked to fill out a self-reporting questionnaire. The questionnaire included the participant’s sex, age, height, body weight, CPR experience, previous CPR training, social status and frequency of regular exercise (>20 min) in a week. The participants were randomly divided into four groups (A, B, C, and D) according to the length of rest period between CC cycles. Each participant performed 2 min CC with different rest periods (Group A-2 min, B-4 min, C-6 min, and D-8 min), therefore a participant in group A, B, C and D needed to do eight, five, four and three times of 2-min CC accordingly during the 30 min period. Each group represented two, three, four, and five personnel, respectively. While performing CCs, the participants were not given feedback on the quality of their CCs. CC assessment was performed by using the ResusciAnne QCPR manikin and ResusciAnne Simulator SimPad version (Laerdal Medical AS, Norway) manikin. CCs more than 65 mm in depth were recorded as 65 mm owing to the limitation of the manikin. The manikins were posted on ground with no metronome.

### Outcomes

Since our trial conducted from April 1, 2015, through December 1, 2016, the 2015 AHA guideline has been published during our trial. We evaluated the quality of CC by using the following variables: 1) a sufficient CC depth ratio that is a number of CCs with adequate (>5 cm) depth per 2 min total CCs time. 2) CC quickness (beats/min) and 3) complete recoil rate (%). Complete recoil rate is a number of CCs with full lift up from the chest per 2 min total CCs.

Among these three variables, we considered a sufficient CC depth ratio (%) as the main outcome of our study because CC depth was clinically proven important factor for the rate of return of spontaneous circulation and survival outcomes [2]. The end-points were measured as percentages and in centimeter by using the Laerdal PC Skill Reporting System.

We compared the differences in the quality of CC between the first cycle and the second to the last cycle. We chose the second to the last cycle for the evaluation because we presumed that the participants could perform better during the last cycle of CCs as a final sprint, which would lead to underestimation of rescuer fatigue.

### Sample size

Sample size calculations were performed using G*Power (Ver3.1.9.2), based on the data from the pilot sample (n = 7) because there were no similar studies before. We calculated minimum required sample of 132 participants testing the null hypothesis that there is no difference in means of the differences of chest compression rates with appropriate depth defined above between the four groups of different chest compression intervals. This sample size is adequate to detect the difference with 80% power and α = 0.05.

We presumed drop rate would be around 5%.

### Randomization

We used a web-based software that is available online (http://www.randomization.com/). The website provides a randomization program to assign the four groups in the ratio of one to one. The participants were blinded to the purpose and outcomes of our study.

### Statistical analysis

The continuous number of our data was presented as median with interquartile range (IQR). The Kruskal-Wallis test was conducted for continuous variables. The Wilcoxon signed-rank test was used as post-hoc analysis when the Kruskal-Wallis test showed statistical significance. The chi-square and Fisher exact test were used to examine the differences between categorical variables. The JMP version 10.0.2 software (SAS Institute, Cary, NC, USA) and SPSS Ver.12 (SPSS Inc., Chicago, IL, USA) were used by three authors (S.Y., J. H., and K.N.) for all the statistical analyses. All the reported probability values were two-tailed, and a p value of <0.05 was considered statistically significant. The primary authors had full access to the data and were responsible for its integrity. All the authors have read and agreed to the manuscript as written.

## Results and discussion

### Participants’ characteristics

**Fig 1:**
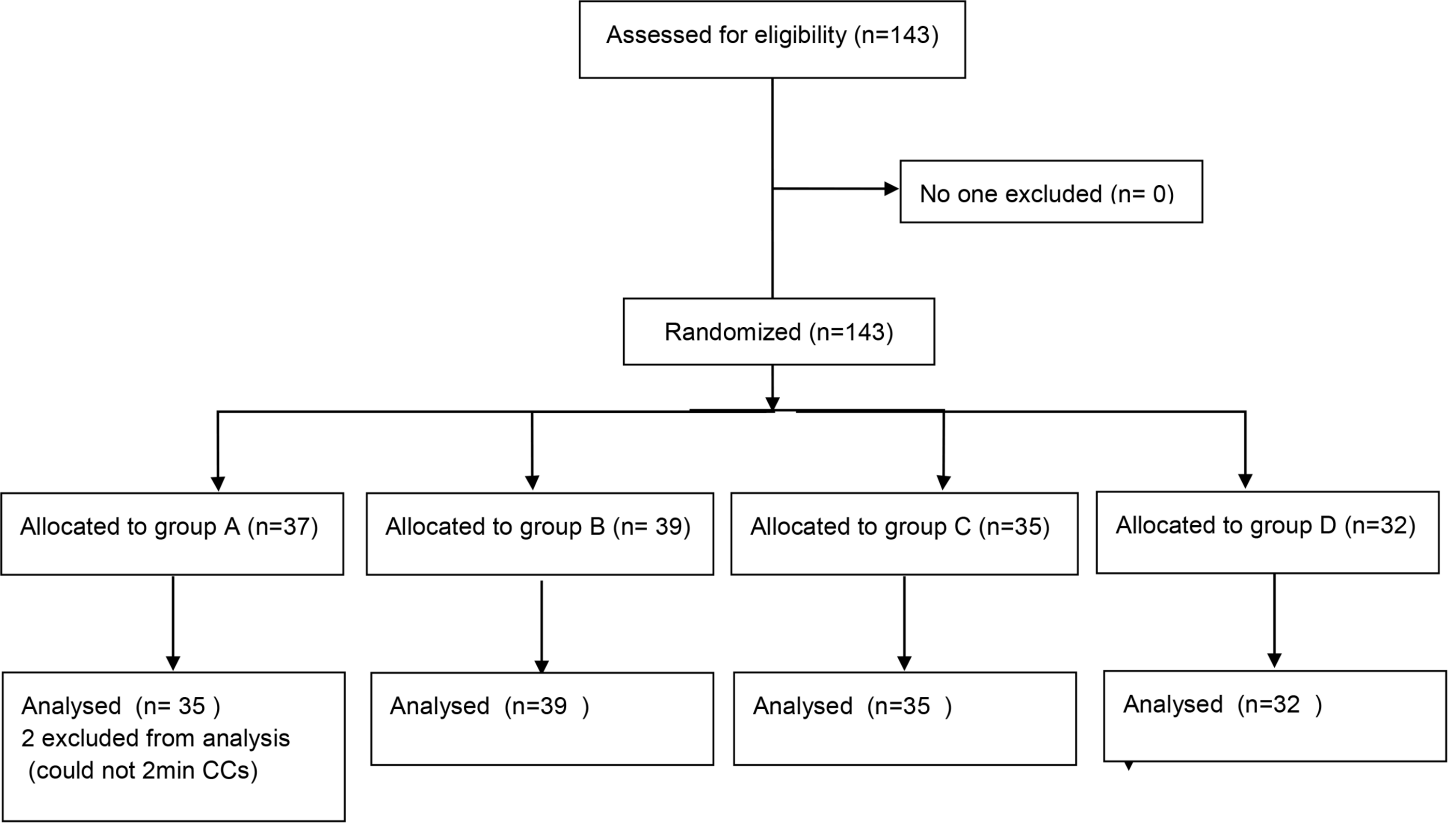
Participant flow chart.

During the study period, we recruited 143 participants (median age 23 years, male 58(40%)). Two participants in group A dropped from this trial because they got too tired to continue CC. Therefore this study involved 141 participants for the analyses. This was more than pre-defined number of participants and therefore we think this study has enough power. We randomly assigned them to one of the four groups. The flow chart was shown in Fig 1. The baseline characteristics of the participants are shown in Table 1. No statistically significant differences in body weight, height, sex, and previous CPR training were observed among the participants of the four groups.

**Table 1:** Participants’ characteristics.

Characteristics	Total (*n* = 141)	Group A	Group B	Group C	Group D	*P-value*
Age—year, median (IQR Q1-Q3)	23 (21–25)	23 (22–26)	23 (22–25)	24 (23–24)	23 (22–24)	0.62
Male gender-no. (%)	58 (41)	15 (42)	15 (38)	15 (42)	13 (40)	0.98
Height-cm,median (IQR Q1-Q3)	163 (158–170)	161 (157–170)	162 (157–170)	165 (158–171)	165 (158–171)	0.72
Weight-kg,median (IQR Q1-Q3)	55 (50–64)	55 (47–63)	53 (48–63)	55 (50–67)	54 (51–65)	0.49
Participant status—no. (%)						0.67
Medical student	70 (50)	17 (49)	20 (51)	19 (54)	14 (44)	
Junior resident	4 (3)	0 (0)	2 (5)	2 (6)	0 (0)	
Nurse	63 (45)	16 (46)	16 (41)	13 (37)	18 (56)	
Staff doctor	2 (1)	1 (3)	1 (3)	0 (0)	0 (0)	
EMS	2 (1)	1 (3)	0 (0)	1 (3)	0	
Any lecture experienced about chest compression -times, median (IQR Q1-Q3)	3 (2–4)	3 (2–3)	3 (2–4)	3 (2–4)	3 (2–4)	0.18
Last lecture-no. (%)						0.37
〜half yr ago	56 (40)	12 (34)	19 (49)	12 (34)	13 (40)	
〜1yr ago	33 (23)	10 (29)	10 (26)	8 (23)	5 (16)	
〜2yr ago	30 (21)	7 (20)	5 (13)	12 (34)	6 (19)	
〜4yr ago	13 (9)	2 (6)	3 (8)	3 (8)	5 (16)	
4yr ago〜	9 (6)	4 (11)	2 (5)	0 (0)	3 (9)	
CPR experience times-no. (%)						
0	130 (92)	33 (94)	37 (95)	32 (91)	28 (88)	0.45
1〜5	9 (6)	1 (3)	2 (6)	2 (6)	4 (13)	
5〜10	1 (1)	1 (3)	0 (0)	0 (0)	0 (0)	
10〜	1 (1)	0 (0)	0 (0)	1 (3)	0 (0)	
20 min exercises in a week-no. (%)						
0	63 (45)	19 (54)	17 (44)	12 (34)	15 (49)	0.98
1	23 (16)	6 (17)	7 (18)	6 (17)	4 (13)	
2	23 (16)	5 (14)	6 (15)	7 (20)	5 (16)	
3	21 (15)	3 (9)	6 (15)	7 (20)	5 (16)	
4	5 (3)	1 (3)	2 (5)	1 (3)	1 (3)	
5〜	6 (4)	1 (3)	1 (3)	2 (6)	2 (6)	

IQR: interquartile range; EMS: emergency medical services; BLS: basic life support; ALS: advanced life support; CPR: cardiopulmonary resuscitation

#### Decline in the quality of CCs depending on decreasing CC personnel

The results of the sufficient CC depth ratio, CC quickness and sufficient recoil ratio of each group in each cycle are shown in Table 2. Even though it was not statistically significant, the median of sufficient CC depth ratio in group A and B gradually decreased over 30 min course as compared to group C and D. In all four groups, CC quickness increased each cycles and recoil rate remained stable overall.

**Table 2:** Sufficient CC depth ratio, CC quickness and complete recoil rate in each cycle over 30 min CPR.

Sufficient (>5cm) CC ratio -%,median (IQR Q1-Q3)									
Group	CC 1	CC2	CC 3	CC 4	CC 5	CC 6	CC 7	CC 8	P—*value*
A	71 (14–97)	54 (6–88)	38 (10–84)	40 (3–91)	21 (2–86)	11 (1–75)	16 (0–92)	15 (2–85)	0.23
B	35 (11–87)	29 (3–86)	30 (1–99)	16 (0–89)	29 (1–98)	N/A	N/A	N/A	0.97
C	52 (23–99)	78 (13–99)	90 (5–100)	61 (5–99)	N/A	N/A	N/A	N/A	0.58
D	49 (6–96)	33 (5–97)	60 (84–97)	N/A	N/A	N/A	N/A	N/A	0.85
CC quickness-times, median (IQR Q1-Q3)									
	CC 1	CC2	CC 3	CC 4	CC 5	CC 6	CC 7	CC 8	
A	119 (109–123)	121 (110–126)	121	120	121	121	123	120	0.93
			(111–128)	(113–127)	(115–131)	(113–131)	(115–131)	(113–129)	
B	117	119	119	120	119	N/A	N/A	N/A	0.62
	(107–120)	(113–124)	(112–126)	(114–125)	(114–127)				
C	118	120	122	123	N/A	N/A	N/A	N/A	0.89
	(112–124)	(116–127)	(116–130)	(117–128)					
D	117	121	123	N/A	N/A	N/A	N/A	N/A	0.22
	(112–123)	(112–126)	(113–129)						
Recoil rate -%,median (IQR Q1-Q3)									
	CC 1	CC2	CC 3	CC 4	CC 5	CC 6	CC 7	CC 8	
A	98 (53–100)	99 (83–100)	98 (89–100)	99 (66–100)	98 (69–100)	99 (94–100)	99 (81–100)	97 (57–100)	0.84
B	99 (94–100)	99 (92–100)	95 (64–100)	99 (86–100)	100	N/A	N/A	N/A	0.89
					(78–100)				
C	99 (92–100)	99 (93–100)	100	100	N/A	N/A	N/A	N/A	0.88
			(76–100)	(91–100)					
D	99 (83–100)	98 (79–100)	98 (67–100)	N/A	N/A	N/A	N/A	N/A	0.74

CC, chest compression; IQR, interquartile range

The main results of our study are shown in Table 3. With the decrease in the CC rest period, a significant decrease in the sufficient CC depth ratio was observed in the second to the last cycle as compared to the first cycle. The median changes were −4%, −3%, 0%, and 0% in groups A, B, C, and D, respectively; the differences were statistically significant (p < 0.01). The decrease was profound for female participants (−13%, −6% 0%, and 0%; p = 0.03). Alternatively, we did not find significant changes for the male participants (0%, 0%, 0%, and 0%; p = 0.4).

**Table 3:** Differences of the outcomes between 1st chest compression cycle and 2nd to the last chest compression cycle.

Primary outcome	Group A	Group B	Group C	Group D	*P-value*
Difference of the sufficient CC ratio-%, median (IQR Q1-Q3)					
Whole	-4 (-36-1)	-3 (-17-0)	0 (-5-4)	0 (-3-4)	<0.01
Male	0 (-9-3)	0 (-13-2)	0 (0–3)	0 (-1-9)	0.4
Female	-14 (-39-1)	-7 (-25-0)	0 (-16-12)	0 (-18-3)	0.03
Secondary outcome					
Difference of the CC quickness-times/min, median (IQR Q1-Q3)					
Whole	-6 (-10—1)	-4 (-10-1)	-5 (-9–1)	-1 (-5-2)	<0.01
Male	-5 (-8—2)	-4 (-8—2)	-5 (-6—1)	-2 (-7-2)	0.49
Female	-7 (-11—1)	-3 (-10—2)	-7 (-10—4)	0 (-4-2)	<0.01
Difference of the sufficient chest-recoil ratio-%,median (IQR Q1-Q3)					
Whole	0 (-6-5)	0 (-1-9)	0 (-2-9)	0 (-4-6)	0.94
Male	0 (-9-9)	0 (-9-10)	0 (0–15)	1 (0–22)	0.68
Female	0 (-4-1)	0 (-1-1)	-1 (-6-1)	0 (-13-3)	0.85

CC, chest compression; IQR, interquartile range

There were statistically significant deference between Group A and C, Group A and D, Group B and C in the sufficient CC depth ratio using the Wilcoxon signed-rank test as post-hoc analysis (P <0.01, P = 0.02, P = 0.02, respectively).

Among the secondary outcomes, we found the significantly higher rate of CC as the CC rest period was shorter. The median changes are 6, 4, 5, and 1 (times/min) in each group respectively (p < 0.01). Again, this change was significant for the female participants (p < 0.01) but not significant for the male participants (p = 0.48). No significant change in sufficient recoil ratio (p = 0.94) was observed.

## Discussion

In this manikin-based randomized parallel trial, we found that rescuers with shorter CC rest period could not maintain CC with adequate depth during 30-min of CPR as compared to those with longer CC rest period. The median changes with regard to a sufficient CC depth ratio decreased by 4% for the two min interval group and by 3% for the four min interval group and these decreases can be 36% and 17%, respectively, according to the 25th percentile.

We consider that these changes can deteriorate the chances of survival, as many previous studies have shown that a subtle change in CC depth affects the outcome and even a slight decrease in CC depth can worsen the rate of return of spontaneous circulation [2, 9–11]. The decrease in sufficient CC ratio was dependent on the rest period; that is, a profound decrease was found in the shorter rest period group. This was consistent with the finding of previous studies, which found a time-dependent deterioration in CC. These studies suggested that rescuer fatigue plays an important role in the decline in CC quality[4, 6, 7]. In fact, several studies proved that rescuer fatigue can negatively affect the quality of CCs, by using physiological markers such as oxygen saturation by pulse oximetry, blood lactate concentration, heart rate, and neuromuscular function[12–14]. Our study also demonstrated that the rate of CC was higher as the CC rest period was shorter, confirming previous study results [2, 15–17]. This finding suggests that faster CC may be inducing rescuer fatigue and leads to poor sufficient CC depth ratio. As CC with pace-making devices such as metronome is known to maintain the adequate speed and depth of CCs[18, 19]. Using such devices during CPR might mitigate the harmful effects of shorter interval in relation to rescuer fatigue as well.

We found a significant decrease in sufficient CC ratio in the female subgroup but found no significant difference among any of the groups in the male subgroup. Previous studies have pointed out that female rescuers have more difficulty in maintaining the quality of CC than male rescuers possibly because of differences in the physical strength[20, 21].

Our study has several strengths. To the best of our knowledge, this is the first randomized controlled trial to determine the appropriate number of personnel to prevent the deterioration of CC quality. Several studies have shown a significant decline in the quality of CCs by consecutive performance due to rescuer fatigue, but the sufficient resting time for maintaining the quality during CPR has been unclear [4]. The finding of our study suggested that at least four personnel in a rescue team with 6 min rest period per person are necessary to maintain the quality of CC during 30 min of CPR.

Our study demonstrates good randomization, and no statistically significant differences were observed among groups A, B, C, and D with respect to age, body weight, height, CPR experience, duration of CPR training and regular exercise habits. In addition, we believe we were able to average the knowledge and skills of the participants through practical training and lectures by using a manikin just before the performance. Therefore, we can conclude that the lack of knowledge or skill had no role in our study results.

Our study also has some limitations. Firstly, most of our participants did not have sufficient clinical experience in CPR. In fact, none of our participants had official AHA BLS/ACLS certification. Thus, we should be careful in generalizing the results for clinically experienced rescuers. However, some previous studies showed that well-trained rescuers such as physicians in the emergency department and EMS also have difficulty maintaining the quality of CC [5, 22]. These findings may imply that well-trained rescuers also need a similar rest period between each CC.

Secondly, the quality of CC was defined based on the 2010 American Heart Association guideline for CPR and emergency cardiovascular care. The 2015 American Heart Association guideline recommends that the depth of CC should be more than five cm and less than six cm. We are unsure how the recommendations could or could not affects our results. Finally, this was a manikin-based study. The differences between the hardness of the chest of the manikin and that of a human must be considered, because this might affect rescuer fatigue.

## Conclusion

According to our research, a 6 min interval after 2 min of CC is vital in order to sustain the quality of CC during a 30-min CPR cycle. Based on the 2015 AHA guidelines for CPR and emergency cardiovascular care[1, 23], rescuers are supposed to change their CC every 2 min during CPR. Hence, our study suggests that the favorable number of personnel for a 30-min CPR with good quality CC during in-hospital cardiac arrest is at least four when the rescue team consists of men and women.

## Supporting information

S1 Table Participants’ characteristics. (XLSX)

S2 Table Sufficient CC depth ratio, CC quickness and complete recoil rate in each cycle over 30 min CPR. (XLSX)

S3 Table Differences of the outcomes between 1st chest compression cycle and 2nd to the last chest compression cycle. (XLSX)

S1 Text Protocol. (DOCX)

S2 Text Questionnaire for participants (Japanese). (DOCX)

S3 Text Questionnaire for participants (English). (DOCX)
