# Supplementary material for: The optimal number of personnel for good quality of chest compressions: A prospective randomized parallel manikin trial
Source: PLoS One. 2017 Dec 21;12(12):e0189412. doi: 10.1371/journal.pone.0189412 (PMC5739419; doi:10.1371/journal.pone.0189412)
Supplement: S1 Text — (DOCX) [file pone.0189412.s004.docx]

| 1. 研究課題名  (study name) | 有効な胸骨圧迫を30分間継続するのに必要なrescuer数の検討  (**The optimal number of personnel for rescuer fatigue and quality of chest compressions: A randomized, crossover manikin trial)** |
| --- | --- |
| 2. 共同研究機関の有無  (Joint research facility) | (1) 共同研究機関 (Joint research facility)  ■① 有り (Yes)  （西山　慶　京都大学 医学研究科初期診療・救急医学分野　講師　倫理委員会あり　）  (Nishiyama Kei: Department of Trauma and Critical Care Center, National Hospital Organization Kyoto Medical Center, Kyoto, Japan)  □ ② 無し  (2) 有りの場合の主任研究機関 (Research representative facility)  　　■ ① 本学 ( Fukui University )  　　□ ② 他の研究機関  　　　　（主任研究機関の名称　　　　　　　　　　　　　　）  　　□ ③ 多施設共同研究に１施設として参加  　　　　（主任研究機関の名称　　　　　　　　　　　　　　）  (3) 有りの場合の本学の役割 (role of Fukui university)  　データ収集など。(Collecting data etc) |
| 3. 研究等の実施場所  (Implementation location of the study) | 福井大学附属病院　シュミレーションセンター　データ収集など  京都大学医学研究科初期診療・救急医学分野　統計処理 (Fukui medical university simulation center) |
| 4. 研究期間  (Period of academic study) | 開始： ■ ① 承認日より (from date of approval; From April 1 2015 )  　　　 □ ② 　　　　年　　月　　日より  終了：　　　　　2016 年　3　月　31　日まで (To December,31,2016) |
| 5. 研究の意義・目的  (The aim of this research) | 既存のBLSガイドラインでは、中断のない胸骨圧迫の重要性が強調されている。その胸骨圧迫の質は、救助者（rescuer）の疲労度に大きく依存することが指摘され、有効な胸骨圧迫を持続させるには複数のrescuerが必要である。  そこで今回の研究の目的は「30分間の有効な胸骨圧迫を維持するのに必要なrescuerの最低人数を検討する」事とした。有効な胸骨圧迫を代表する測定因子としてPrimary Outcomeを「圧迫の速さ（回/分）」、「圧迫の深さ（cm）」などに設定・測定し、30分間経過時点もしくは30分間の推移を評価する。その結果、院内CPRの際には有効な胸骨圧迫を、30分間継続するのに最低何人をrescuerとして集める事が求められるかを検討する。  (There are many guidelines and researches to emphasis how important continuous chest compressions are for good neurological outcome of CPR.  The quality of chest compression is strongly related to rescuer’s fatigue. Hence, calling for many rescuers is necessary to maintain the quality of chest compression. But medical resources are limited.  The aim of our study is to determine the optimal number of personnel needed for 30-min CPR in a rescue-team. If we can find the smallest number, we will be able to save limited medical resources and guarantee of the quality.) |
| 6. 研究方法  (Methods of the study) | 研究デザインの型 ( Type of study)  ・マネキンを使用したシミュレーション研究  ・ランダム化比較交差試験  (**A randomized, crossover manikin trial)**  ①2010年　AHA　BLSガイドラインでは胸骨圧迫は2分間隔で交代するのが望ましいとされている。これに従い、  ・被験者を  　　　O群：30分間継続して胸骨圧迫を行う群  (30分間の胸骨圧迫を1人で行う場合に該当)  A群：2分間胸骨圧迫⇒2分間休憩　を30分間繰り返す群  (30分間の胸骨圧迫を２人で行う場合に該当)  B群：2分間胸骨圧迫⇒4分間休憩　を30分間繰り返す群  (30分間の胸骨圧迫を３人で行う場合に該当)  C群：2分間胸骨圧迫⇒6分間休憩　を30分間繰り返す群  (30分間の胸骨圧迫を４人で行う場合に該当)  　 D群：2分間胸骨圧迫⇒8分間休憩　を30分間繰り返す群  (30分間の胸骨圧迫を５人で行う場合に該当)  　の5郡にランダム割り付けにより分ける。  （上記のグループ数はPilot studyの結果　３つ程度に減る可能性がある）  ②其々のグループに30分間のCPRを行ってもらい、それぞれの群の胸骨圧迫の質を計測する。  胸骨圧迫の質は  １）胸骨圧迫の深さ5㎝以上行えた回数/5㎝以下の回数の割合  　　２）2分間にわたり胸骨圧迫の回数/分＞100回以上を維持できたか　その回数  　　３）正しくRecoil（胸骨圧迫の解除）ができたか　Recoil率の計測  の3つで評価する。  　Primary outcomeは１）とする。  (After a 30-min lecture and practical training session using manikins according to the 2010 AHA guidelines, participants were asked to fill out a self-reporting questionnaire. The questionnaire included the participant’s sex, age, height, body weight, CPR experience, previous CPR training, social status, educational background, frequency of regular exercise (>20 min) in a week, and ethnicity/religion.  The participants were randomly divided into 4 groups (A, B, C, and D) according to different CC rest periods (2, 4, 6, and 8 min). Each group with different rest periods represented 2, 3, 4, and 5 personnel, respectively. All the participants performed CCs for 30 min with these different rest periods (2, 4, 6, and 8 min).  While performing CCs, the participants were not given feedback on the quality of their CCs. CC assessment was performed by using the ResusciAnne QCPR manikin and ResusciAnne Simulator SimPad version (Laerdal Medical AS, Norway) manikin. CCs more than 65 mm in depth were recorded as 65 mm owing to the limitation of the manikin. The manikins were posted on ground.  According to the 2010 AHA recommendations, we evaluated the quality of CC by using the following variables: 1) number of CCs with a depth of more than 5 cm (times/2 min), 2) a CC ratio with an appropriate rate of at least 100/min and less than 120/min (%), and 3) complete recoil rate (%). Among these 3 variables, we considered a sufficient CC depth ratio (%) as the main outcome of our study because CC depth was the most important factor for the rate of return of spontaneous circulation and survival outcomes. The end-points were measured as percentages and in centimetre by using the Laerdal PC Skill Reporting System.  We compared the differences in the quality of CC between the first cycle and the second to the last cycle. We chose the second to the last cycle for the evaluation because we presumed that the participants could perform better during the last cycle of CCs as a final sprint, which would lead to underestimation of rescuer fatigue.)  Following picture is for reviewers to understand our research design,   \| **Min.** \| 2 \| 4 \| 6 \| 8 \| 10 \| 12 \| 14 \| 16 \| 18 \| 20 \| 22 \| 24 \| 26 \| 28 \| 30 \| \| --- \| --- \| --- \| --- \| --- \| --- \| --- \| --- \| --- \| --- \| --- \| --- \| --- \| --- \| --- \| --- \| \| A(2 personnel) \| CPR1 \| Rest \| CPR2 \| Rest \| CPR3 \| Rest \| CPR4 \| Rest \| CPR5 \| Rest \| CPR6 \| Rest \| CPR7 \| Rest \| CPR8 \| \| B(3 personnel) \| CPR1 \| Rest \| Rest \| CPR2 \| Rest \| Rest \| CPR3 \| Rest \| Rest \| CPR4 \| Rest \| Rest \| CPR5 \| Rest \| Rest \| \| C(4 personnel) \| CPR1 \| Rest \| Rest \| Rest \| CPR2 \| Rest \| Rest \| Rest \| CPR3 \| Rest \| Rest \| Rest \| CPR4 \| Rest \| Rest \| \| D(5 personnel) \| CPR1 \| Rest \| Rest \| Rest \| Rest \| CPR2 \| Rest \| Rest \| Rest \| Rest \| CPR3 \| Rest \| Rest \| Rest \| Rest \|   We compared the quality of chest compressions between CPR1 and the 2^nd^ to the last CPR term  ３）解析法はノンパラメトリック検定 (Statistical analysis)  The Kruskal-Wallis test  ４）調整すべき変数・交絡因子として ( variable , confounding factor)  　・年齢　・身長　・体重　・性別　・BLSの授業を今まで何回うけたか　・最近のBLSの授業の時期　・Bystander CPRを今までに経験した事があるか　・何回実際のCPRを経験したか　・研究の目的の覚知率　・人種　・最高学歴　・職種　・BLS、ACLSなどのCertificationを保持しているか　などを事前にアンケートにて聴取する。  ( age, height, weight, status, experienced BLS time, Last BLS course, ALS/BLS certification, religion,  Exercise time) |
| 7. 研究対象者(被験者)及びその人数，選定方針  Participants | 福井大学医学部附属病院　　1,2年目　研修医　　　約30 人  　 1年目　看護師1年　　約80人/年  福井大学医学部医学科  　　　　　　　　　5年生（ポリクリ） 　約100人　/年  　　　　　　　　　4年生　　　　　　　　約100人 /年  　　　　　　　　　　　　　　　　　　　　　　　　　　合計300人  上記の正確な数は2015年3月末まで確定されないため、概算。  Fukui medical university : junior resident 30 person/year  EMS students and nurse student 80 person/year  Medical student 100/year  Based on our data from a small sample, we estimated effect size and calculated the sample size of 136 to have a two-sided significance level of 5% and 80% power. |
| 1. 研究参加に伴う利益及び不利益   (conflict　of interest) | (1) 研究対象者(被験者)に期待される利益　（benefits for participants through this research）  □ ① 直接的な利益は期待できない  ■ ② 直接的な利益が期待できる (direct benefits are expected)  （医師、看護師として勤務するのに必要な技術の一つである正しい胸骨圧迫の行い方が理解できる。それに付随した心肺蘇生法の知識習得。）  (Because chest compression is a basic procedure as a medical stuff, so participants can understand how to do correct chest compression through our research)  (2) 研究の成果によって将来的にもたらされうる利益 (Social benefits of this research)  ①rescuerの疲労度を超えた小人数での心肺蘇生が減少し、患者の蘇生率向上につながる可能性がある。  ②院内発生の心肺蘇生に必要なrescuerの最低人数が算出され、医療資源の有効利用が可能になる。  (We can guarantee the quality of chest compression. This might be related to good outcome of victims)  (We can save medical resources with the smallest number of chest compression staffs)  (3) 研究対象者(被験者)に起こり得る危険や必然的に伴う心身の不快な状態に対する具体的配慮  (Consideration of participant`s possible risks)  O群の30分間継続して行うグループに分類された被験者は、自己の体力に応じて途中で胸骨圧迫を中断することができる。  　A～D群においては体力的な問題は生じないと予想される。  (All participants have a right to stop chest compression if they are worn out. So, we think there are little risk for participants. |
| 1. 研究対象者(被験者)に健康被害が生じた場合の補償等の具体的な措置   (Compensation methods if something bad happen to participants) | (1) 研究の種類 (kind of study)  □ ① 介入を伴う研究であって，医薬品又は医療機器を用いた予防，診断又は治療方法に関するもの（体外診断を目的とした研究を除く）  *※ 補償のための保険その他の必要な措置について，事前に十分な説明を行い，研究対象者(被験者)の同意を受けなければならない。（その他必要な措置は，例えば，健康被害に対する医療の提供及びその他の物又はサービスの提供をいう。）*  ■ ② それ以外の介入を伴う研究（体外診断を目的とした研究を含む）(Manikin study)  *※ 補償の有無を説明する必要がある。*  □ ③ 観察研究であるが，試料等の採取に侵襲性を伴うもの  *※ 補償のための保険その他の必要な措置の有無を，研究対象者(被験者)に十分説明する必要がある。*  (2) 補償等の具体的な措置  ■ ① 有害事象は生じ得ない ( There is no risk to happen something bad for participants)  　　　（今回の研究により健康被害が生じる可能性はすくない。  O群の体力消耗には十分配慮し、途中で胸骨圧迫を中断することが可能とする。　）  □ ② 保険診療により対応  □ ③ 民間の損害保険により補償  　　　　（損害保険会社名：　　　　　　　　　　　　　　　　　　　　　）  □ ④ 保険以外の対処方法により対応  　　　　（具体的に：　　　　　　　　　　　　　　　　　　　　　　　　）  □ ⑤ その他  　　　　（具体的に：　　　　　　　　　　　　　　　　　　　　　　　　） |
| 1. 個人情報の保護   Protection of personal information | (1) 個人情報管理者（　救急部　　医員　山中　俊祐）( a responsible person : Yamanaka Syunsuke)  (2) 匿名化について ( anonymization)  　　□ ① 連結不可能匿名化　 ■ ② 連結可能匿名化　( anonymization in a linkable fashion)  □ ③ 匿名化しない  　　②③の場合のその理由  　　　■ ① 解析結果を研究対象者(被験者)に知らせる可能性があるため  (Because we need to feed back for improving participant`s chest compression)  　　　□ ② 追跡データと関連させる必要があるため  　　　□ ③ その他  　　　　　（具体的に：　　　　　　　　　　　　　　　　　　　　　　　）  (3) 連結可能匿名化の個人名と記号の連結表や，匿名化されていないデータの保管場所・方法について  ■ ① 他のコンピュータから独立したコンピュータを使用し，外部記憶媒体に記録させ，その記憶媒体は，鍵をかけて厳重に保管  (We use an isolated PC to save the data with external storage. We place the storage in the locked room)  □ ② 筆記による原簿として，鍵をかけて厳重に保管  　　□ ③ その他  　　　　（具体的に：　　　　　　　　　　　　　　　　　　　　　　　　） |
| 1. 研究対象者(被験者)に理解を求め同意を得る方法(How to get informed consent) | (1) 研究対象者(被験者)に理解を求め同意を得る方法について  　　■ ① 文書によるインフォームド・コンセントを受ける  　　　　　（説明書及び同意書を添付）  (To get an informed consent through a document)  □ ② インフォームド・コンセントは受けないが，研究の目的を含む研究の実施についての情報を公開し，また，研究対象者(被験者)となる者が研究対象者(被験者)となることを拒否できるようにする  （情報公開の方法：　　　　　　　　　　　　　　　　　　　　　）  （研究対象者(被験者)となることの拒否を保障する方法：　　　　）  □ ③ インフォームド・コンセントは受けないが，研究の目的を含む研究の実施についての情報を公開する  （情報公開の方法：　　　　　　　　　　　　　　　　　　　　　）  　　□ ④ インフォームド・コンセントを得ないで研究を行う  　　　　（その理由：　　　　　　　　　　　　　　　　　　　　　　　　）  　　□ ⑤ その他  　　　　（具体的に：　　　　　　　　　　　　　　　　　　　　　　　　）  (2) 代諾の有無 ( legal representative)  □ ① 代諾有り（研究対象者(被験者)が未成年者の場合，成年者でも十分な判断力の無い場合，又は病名に対する配慮が必要な場合等）  1) 当該研究の重要性：  2) 研究対象者(被験者)の参加が研究を実施するに当たり必要不可欠な理由：  3) 代諾者の選定方針：  ■ ② 代諾無し (There is no need to prepare a legal representative) |
| 12. 研究期間終了後の試料の取扱い  (The handling of the data after the study) | ■ ① 保存する (to save)  1) 試料等の名称(name)：　胸骨圧迫の質に関する資料 (data of chest compression quality)  2) 試料等の管理責任者：救急部　医員　山中俊祐 ( a responsible person : Yamanaka Syunsuke)  3) 試料等の保管場所( place to save data)  ：　責任者の外部取り付けハードディスク ( External device of a responsible person PC)  4) 試料等の保存期間( data retention period) ：5年間（2021年3月31日まで）(5 years)  5) 保存が必要な理由 (reason of the data retention)：資料の解析のため (to statistical analysis)  6) 研究対象者(被験者)から得た同意の内容（将来にわたって試料を解析する場合はその旨を対象者に説明し同意を得ること）(methods of getting an informed consent)  ：同意書にて同意取得 (By documents)  7) 匿名化の方法 (Methods of anonymization)  ■ ① 連結可能匿名化 ( anonymization in a linkable fashion)  （その理由：被験者に今回評価した胸骨圧迫の質をフィードバックする可能性があるため。被験者はそれを通して、より質の高い胸骨圧迫を行えるようになる。）  ( Because we need to feed back for improving participant`s chest compression)  □ ② 連結不可能匿名化  □ ② 廃棄する  □ ① 人体から採取された試料  （廃棄の方法：　　　　　　　　　　　　　　　　　　　　　）  □ ② 診療情報  （廃棄の方法：　　　　　　　　　　　　　　　　　　　　　）  □ ③ その他の資料  （廃棄の方法：　　　　　　　　　　　　　　　　　　　　　） |
| 13. 研究開始前に人体から採取された試料等の利用  (Use of sample got from human body) | □ ① 研究開始前に人体から採取された試料等を利用する  □ ① 利用に関して改めて研究対象者(被験者)等から同意を得る  （説明書及び同意書を添付）  □ ② 研究対象者(被験者)等から同意を得ない  □ ① 当該試料は匿名化されている  □ ② 当該試料は既に他の研究への利用に関して研究対象者(被験者)から同意が得られており，研究の実施について利用目的を含む情報を公開する  □ ③ 同意を得ることが困難であるので，研究の実施について試料の利用目的を含む情報を公開し，研究対象者(被験者)となることを拒否できるようにする  □ ④ 公衆衛生の向上のために特に必要がある場合であって，研究対象者(被験者)の同意を得ることが困難である  ■ ② 研究開始前に人体から採取された試料等を利用しない (Not applicable) |
| 14. 他の機関等の試料等の利用  (data usage from other facilities) | (1) 他の機関等からの試料等の提供 (data usage from other facilities)  □ ① 提供を受ける  試料等の内容：  必要性：  ■ ② 提供を受けない (No)  (2) 他の機関等への試料等の提供 (Sending date got at this facility to other one)  ■ ① 提供する (yes: low data for analysis)  試料等の内容：　生データ  必要性：データ編集のため   - ② 提供しない |
| 15. 研究成果の公表  (announce methods of the research) | ■ ① 研究対象者(被験者)を特定できないようにした上で，学会や学術雑誌で発表する  (We will submit a paper from this study to a journal or present at international conferences.  □ ② 公表は予定していない  □ ③ その他（　　　　　　　　　　　　　　　　　　　　　　　　　　 ） |
| 16. 使用する研究費  (research funds) | ■ 自己収入　(By myself)　 □ 科学研究費補助金　　 □ 厚生労働科学研究費補助金  □ その他の公的研究費（　　　　　　　　　　　　　　　　　　　　　　）  □ 奨学寄附金・研究助成金  □ 共同研究費（　　　　　　　　）　　□ 受託研究費（　　　　　　　 ）  □ その他（　　　　　　　　　　　　　　　　　　　　　　　　　　　　） |
| 17. 本研究と企業・団体との関わり  (Any relationship to companies) | ■ ① 本研究に企業等は関与しない (none)  □ ② 共同研究として実施（相手先：　　　　　　　　　　　　　　　　）  □ ③ 受託研究として実施（相手先：　　　　　　　　　　　　　　　　）  □ ④ 本研究に関与する企業等はあるが，共同研究・受託研究として実施しない  　　　（相手先及び関与の具体的内容：　　　　　　　　　　　　　　　） |
| 18. 起こりうる利害の衝突*＊*  （該当するもの全てにチェック）  (possible risks for participants) | □ ① 研究実施責任者及び研究者等が，本研究に関与する企業等から，本研究への関連の有無に関わらず共同研究費，受託研究費，委任経理金（奨学寄附金）等の研究資金を受け入れている。  □ ②本研究に関与する企業等との間に，本研究への関連の有無に関わらず機器や消耗品等の提供，依頼試験・分析，研究員の受入等がある。  　　（具体的に：　　　　　　　　　　　　　　　　　　　　　　　　）  □ ③ 研究実施責任者及び研究者等が，本研究に関与する企業等との間に，役員・顧問等の非常勤を含む雇用関係，研究資金以外の謝金・報酬・給与の支払い等の経済的利益，知的財産権（特許や実用新案等）の共有・実施許諾・譲渡がある。又は，当該企業の株式（未公開株・ストックオプションを含む）を所有している。  　　（個人情報を保護できる範囲で具体的に：　　　　　　　　　　　）  □ ④ 研究実施責任者及び研究者等の，１親等以内の親族が，本研究に関与する企業等との間に，研究費の受入，報酬・給与等の経済的利益，経営関与，知的財産権（特許や実用新案等）の共有・実施許諾・譲渡がある。又は，当該企業の株式（未公開株・ストックオプションを含む）を所有している。  　　（個人情報を保護できる範囲で具体的に：　　　　　　　　　　　）  □ ⑤ 本研究の実施によって，本務である教育・研究・診療等に支障をきたす可能性がある。  　　（具体的に：　　　　　　　　　　　　　　　　　　　　　　　　）  ■ ⑥ 利害の衝突は起こり得ない ( There is no risk for participants) |

別紙様式第２号
