# Supplementary material for: The optimal number of personnel for good quality of chest compressions: A prospective randomized parallel manikin trial
Source: PLoS One. 2017 Dec 21;12(12):e0189412. doi: 10.1371/journal.pone.0189412 (PMC5739419; doi:10.1371/journal.pone.0189412)
Supplement: S2 Text — (DOCX) [file pone.0189412.s005.docx]

事前アンケート　通し番号

　　月　　日　　　　　　　　　　　所属（医師　看護師　医学科　看護科　救急救命士）

学籍番号　　　　　　　　　　　　　　　　もしくはイニシャル

以下の質問に答えてください。

**このデータは個人が特定されないように加工され、研究以外の目的で使用・公開されることはありません**。

CPR＝Cardiopulmonary resuscitation 心肺蘇生処置

BLS＝Basic life support 一次救命処置

ACLS＝Advanced cardiac life support　高次救命処置

１）性別　職種・所属を教えてください

　　（男性　・　女性　）　（研修医　看護師　救急救命士　医学科学生　看護科学生　）

２）年齢を教えてくさい

　　（　　　　）　歳

３）身長・体重を教えてください

（身長　　cm　　体重　　kg）

４）BLS、ACLSなど心肺蘇生の講習に、今まで何回参加した事がありますか？

（ない　１回　２回　３回　４回　５回以上）

５）BLS、ACLSなど心肺蘇生の講習を最後にいつ受講しましたか？

　　　（半年以内　半年~1年以内　1~2年以内　2~3年以内　3~4年以内　5年以上前）

６）BLS、ACLSのインストラクターですか？

　　　（　はい　・　いいえ　）

７）実際に意識がない患者さん対象にCPRを行った経験はありますか？

　（　はい　・　いいえ　）

８）実際に意識がない患者さん対象にCPRを今までに何回行ったことがありますか？

　　　（経験なし　・　　回程度行ったことがある）

９）学歴を教えてください

（　高卒・大学生・異なる大学を卒業後の本学生・異なる大学院を卒業後の本学生　）

１０）宗教を教えてください

　【特になし　・仏教　・キリスト教　・その他（　　　　　）】

以上　ご協力ありがとうございました。
