# Supplementary material for: The optimal number of personnel for good quality of chest compressions: A prospective randomized parallel manikin trial
Source: PLoS One. 2017 Dec 21;12(12):e0189412. doi: 10.1371/journal.pone.0189412 (PMC5739419; doi:10.1371/journal.pone.0189412)
Supplement: S3 Text — (DOCX) [file pone.0189412.s006.docx]

事前アンケート　通し番号 (questionnaire for participants)

　　月　　日　(month---------- day------------)

所属（医師　看護師　医学科　看護科　救急救命士）

Occupation / department (doctor, nurses, resident, medical student, nursing student, EMS)

学籍番号　　　　　　　　　　　　　　　　もしくはイニシャル

Student I.D. 　　　　　　　　　 Initial ( )

以下の質問に答えてください。

**このデータは個人が特定されないように加工され、研究以外の目的で使用・公開されることはありません**。

CPR＝Cardiopulmonary resuscitation 心肺蘇生処置

BLS＝Basic life support 一次救命処置

ACLS＝Advanced cardiac life support　高次救命処置

Answer these questions below. This will be anonymized and will not to use other purposes except for research.

１）性別　職種・所属を教えてください

　　（男性　・　女性　）　（研修医　看護師　救急救命士　医学科学生　看護科学生　）

Answer your Sex, occupation and department

(Male, Female) (Doctor, nurses, resident, medical student, nursing student, EMS)

２）年齢を教えてくさい How old are you ?

　　（　　　　）　歳 ( ) years old

３）身長・体重を教えてください What is your weight/ height ?

（身長　　cm　　体重　　kg）

４）BLS、ACLSなど心肺蘇生の講習に、今まで何回参加した事がありますか？

Have you ever participated in BLS/ACLS, Chest compression lecture and any study opportunities about BLS? How many times is it?

（ない　１回　２回　３回　４回　５回以上）

(Never, 1, 2, 3, 4, more than 5)

５）BLS、ACLSなど心肺蘇生の講習を最後にいつ受講しましたか？

When was the last time you took the lecture?

　　　（半年以内　半年~1年以内　1~2年以内　2~3年以内　3~4年以内　5年以上前）

(less than half year, 0.5〜1 year, 1~2, 2~3,3~4, more than 5)

６）BLS、ACLSのインストラクターですか？

Are you an instructor of BLS, ACLS that are certified by AHA?

　　　（　はい　・　いいえ　）( yes / no )

７）実際に意識がない患者さん対象にCPRを行った経験はありますか？

Have you ever performed CPR for a real patients / a body ?

　（　はい　・　いいえ　）

８）実際に意識がない患者さん対象にCPRを今までに何回行ったことがありますか？

How many is it ?

　　　（経験なし　・　　回程度行ったことがある）(Never / times)

９）学歴を教えてください

（　高卒・大学生・異なる大学を卒業後の本学生・異なる大学院を卒業後の本学生　）

Write down your final educational background . ( )

１０）宗教を教えてください

　【特になし　・仏教　・キリスト教　・その他（　　　　　）】

What is your religious belief? ( )

以上　ご協力ありがとうございました。

Now questionnaire is over, thanks.
